# Navigating online health information: empowerment vs. misinformation

**DOI:** 10.3389/fdgth.2025.1555290

**Published:** 2025-07-24

**Authors:** Alan Silburn

**Affiliations:** School of Medicine, Western Sydney University, Campbelltown, NSW, Australia

**Keywords:** internet-based patient education, health literacy, misinformation, accessibility, empowerment, credible resources

## 1 Introduction

Since the beginning of medicine, patient education has progressively transitioned from the indisputable advice and direction of the practitioner to the introduction of publicly available printed medical literature, to the current landscape whereby every facet of the world's medical knowledge can be instantly digitized, interpreted, and illustrated to any patient that seeks it via internet-based devices. However, with such a breadth of information available, false or misleading interpretations, incorrectly published data, or unsubstantiated claims make navigation of internet-based patient education challenging (1). Nevertheless, with the right direction and guidance, internet-based patient education can have profoundly positive impacts on patients' health. This commentary discusses the advantages and challenges of internet-based patient education by examining four common contexts: the information is accurate and applicable; the information is inaccurate and perceived as applicable; the information is inaccurate and non-applicable; and the inability to access information.

## 2 Discussion

### 2.1 Accurate and applicable information

Most patients have a range of information freely available online that can be used to educate them on their physical health, such as their disease status, prognosis, complications, and self-management options, in addition to their mental and spiritual health, including information on social support services, alternative medicines, and illness-specific counseling (2). This information may affect the patient's experience of illness and their subsequent interactions with their healthcare providers.

In a positive context, providing the information is both correct and applicable, an increase in consumer knowledge has been linked to improved health outcomes as patients become increasingly engaged in the decision-making process. This allows for greater health status awareness, increased understanding and adherence to medical instructions, and increased confidence in the self-management of their healthcare (2). An Australian study found that more than 33% of adult patients engaged in internet-based searches about their presenting problem before attending the emergency department, especially among younger and e-health literate patients (3). The authors concluded that these searches had a mostly positive impact on the patient–doctor interaction and that patients were less likely to question the diagnosis or treatment (3).

Unfortunately, some patients reported contrastingly negative effects, as search results increased their anxiety attributed to learning about their possible prognosis or likely required treatments (3). Although the information was both correct and applicable, it is a consideration that the oversimplification of some medical terminology can lead to an inadvertent focus on complications rather than positive outcomes. In the event a poor health outcome does occur, patient support group sites are available for patients to educate themselves on an array of personal, psychological, and social interventions that may be beneficial to their situation and provide means of access to such services (4).

### 2.2 Inaccurate but perceived-as-applicable information

Challenges arise when patients become misdirected or misinterpret information that they, in turn, perceive as appropriate to their setting. For example, this occurs when responding to a patient who requested unsupported treatments or unnecessary tests for a similar condition after reading gray literature. Gray literature includes unregulated blogs, forums, and non-peer-reviewed sources that may contain persuasive but unreliable claims. Infrequently, acquired information can lead to conflict if the patient values the internet-derived information above that of the doctor. Sadly, this can potentially cause them to disregard any given health advice (3).

Although there is concern about the misdirection and misinterpretation of health resources, the enthusiasm of a patient's quest for the best healthcare should not be undermined, often patronizingly referred to as using “Dr. Google” (5). Instead, demonstrating appropriate evidence-based practice searching and providing guidance toward credible resources can clearly define why their request cannot be fulfilled and will encourage a patient–doctor relationship built on respect and humility (3). Frameworks such as the Currency, Relevance, Authority, Accuracy, Purpose (CRAAP) test can help patients critically assess online information. Trusted platforms include the National Library of Medicine, Healthdirect, and the Mayo Clinic.

### 2.3 Inaccurate and non-applicable information

Like Google, YouTube contains a wide variety of unverified content that may promote unhealthy behaviors and activities (6). Unfortunately, the video platform's algorithms are characterized by popularity-driven data rather than expert review or assessment. This exposes the user to videos that can be perceived as reliable information about their condition, yet remain unsubstantiated, fundamentally flawed, and non-applicable in context. Although clinicians agree that learning medical theory is possible from such a platform, it is emphasized that content must be verified for accuracy and currency before adopting it as an educational resource (7).

Patients may internalize misleading narratives, and confirmation bias can exacerbate this, especially if the content aligns with their pre-existing beliefs. Clinicians can address this by asking where patients found their information, then collaboratively reviewing the material to identify inaccuracies and clarify misunderstandings. This can improve the therapeutic alliance while maintaining the patient's dignity and curiosity.

### 2.4 Lack of access to online health information

Internet-based patient education is popular for a range of reasons outlined; however, barriers such as a lack of information technology skills, lack of computer or device access, poor internet infrastructure, physical or mental disability, or concern for identity security are all challenges identified by some patients (4). This reveals a key variable driving health disparity, as these patients are unable to access both relevant information promoting positive health outcomes or the biopsychosocial support networks to assist in limiting health deterioration.

For this setting, the practitioner should ensure that internet-based patient education is appropriate for the individual patient and that access and navigation can be facilitated. Supplementing digital resources with printed materials, interpreter services, or one-on-one education can help mitigate inequity. On a systemic level, investment in public access points (e.g., libraries, community centers) and digital literacy programs is essential.

### 2.5 Strengthening clinical practice through actionable strategies

To support patients navigating the digital health landscape, clinicians can adopt a range of simple, moderate, and large-scale strategies tailored to their practice setting and patient population.

Simple strategies involve point-of-care actions that can be implemented immediately with minimal resources. Clinicians can recommend trustworthy websites during consultations and follow up with brief discussions that address any online material patients may have read. Asking, “Have you looked anything up about this?” validates the patient's initiative and opens the door for clarification. Displaying short evaluation checklists (e.g., assessing accuracy, source, and purpose) in exam rooms or digital kiosks can subtly prompt critical thinking without being prescriptive.

Moderate strategies include creating previsit or postvisit communications that address common misconceptions or direct patients to clinic-curated content. Medical reception staff or nurses can be trained to triage questions about online findings, referring complex queries to the clinician. Practices could also introduce a brief internet-use questionnaire as part of patient intake to identify those likely to benefit from tailored digital guidance. This promotes efficiency while allowing clinicians to prioritize time with high-risk groups.

Large-scale strategies involve longer-term investments. Clinical teams can collaborate with patient advocacy groups to codesign leaflets or videos that reflect lived experiences and reinforce accurate information. In addition, local health districts can develop shared “approved” digital resource libraries accessible across general practice software, telehealth systems, and community hubs. Efforts to align digital patient education with national health campaigns (e.g., immunization, cancer screening) can amplify messaging and improve reach.

Ultimately, integrating these strategies into routine practice affirms the patient's right to accessible, high-quality information while reinforcing the clinician's role as a trusted guide. The goal is not to replace online learning but to shape it, ensuring that curiosity leads to confidence.

## 3 Conclusion

In conclusion, the world's medical knowledge can be instantly digitized, interpreted, and illustrated to any patient that seeks it, from historic literature searches on Google to modern displays of videos and lecture series on YouTube. Whichever the medium, the practitioner needs to be attentive yet humble when addressing challenges such as false or misled interpretations, incorrectly assessed data, or unsubstantiated requests made by patients following educational misadventures. Provided with the right direction and guidance, and assuming the information is accessible, accurate, and applicable, internet-based patient education can have profoundly positive impacts on patients' health outcomes. Clinicians must therefore lead in this space with clear, empathetic, and evidence-based responses that help patients safely navigate their digital health journeys.
